# The Mediation Role of Sleep Disturbances between Vitamin D and Depressive Symptoms: A Cross-Sectional Study

**DOI:** 10.3390/brainsci13111501

**Published:** 2023-10-24

**Authors:** Lihua Yao, Mianmian Chen, Nan Zhang, Simeng Ma, Xinhui Xie, Shuxian Xu, Zhaowen Nie, Wei Wang, Enqi Zhou, Shunsheng Xu, Shenhong Weng, Hexiang Chen, Dan Xiang, Zhongchun Liu

**Affiliations:** 1 Department of Psychiatry, Renmin Hospital of Wuhan University, Wuhan 430060, China; rm001885@whu.edu.cn (L.Y.); cmmian08@163.com (M.C.); 15071459916@163.com (N.Z.); simeng.ma@foxmail.com (S.M.); xxh.med@gmail.com (X.X.); shuxianxu@outlook.com (S.X.); nzw0525@163.com (Z.N.); wangweiwhu@foxmail.com (W.W.); zeqzeq1998@163.com (E.Z.); doctorxu999@21cn.com (S.X.); wshcj@hotmail.com (S.W.); xiangdannuli@163.com (D.X.); 2 Department of Anesthesiology, Renmin Hospital of Wuhan University, Wuhan 430060, China; chx163yx@163.com; 3 Taikang Center for Life and Medical Sciences, Wuhan University, Wuhan 430072, China

**Keywords:** sleep, depression, vitamin D, mediation, PHQ-9, HAMD-17

## Abstract

Depression and sleep disturbances are highly prevalent health problems that have been suggested to be associated with vitamin D deficiency. This study investigated whether sleep disturbances affect the association between vitamin D and depressive symptoms. A total of 425 patients with depression were included in this study. Spearman correlation coefficients were chosen to assess the relation between vitamin D concentrations and depressive symptomatology (according to the PHQ-9 and HAMD-17 scores). The GLM Mediation Model in the Medmod module for data analysis in Jamovi 2.2.5 was used to analyze the mediation models for sleep disturbances. Vitamin D concentrations were significantly correlated with PHQ-9 and HAMD-17 scale scores. In addition, item 3 was suggested to have a mediating effect between vitamin D and depressive symptoms in the mediating model of PHQ-9, and item 4 was suggested to have a mediating effect between vitamin D and depressive symptoms in the mediating model of HAMD-17. Sleep disturbances (especially difficulty falling asleep) are mediators between vitamin D and depressive symptoms, suggesting that increasing vitamin D levels at the right time to regulate sleep disturbances may improve depression symptoms, yet further research is necessary.

## 1. Introduction

Depression is a common mental disorder with high disability and suicidality [1]. The burden of depression ranks second, being only surpassed by that of cardiovascular disease. The World Health Organization Global Burden of Disease Survey estimates that by 2030, depression will rank first in the number of disability cases experienced by sufferers [2]. The pathogenesis of depression still remains unclear, and approximately 30% of people treated for depression do not achieve remission [3]. Many patients avoid or discontinue treatment because of stigma or fear of side effects. For example, only 0.5 percent of patients suffering from depression in China receive adequate treatment [4]. Consequently, looking for an appropriate and effective treatment for depression is particularly important.

Vitamin D is a fat-soluble vitamin also known as a steroid hormone. It is involved in various human life processes [5], including calcium and phosphorus balance, cell proliferation and differentiation, inflammation and immunity, nervous system regulation, and more. Vitamin D works by binding to vitamin D receptors (VDR), which are expressed in many tissues and organs, including areas of the brain associated with mood and behavior [6]. Recent studies have found a strong link between vitamin D and mood disorders. For example, studies have shown that depressed people have significantly lower levels of vitamin D than the general population (30 ng/mL) [7,8,9,10,11] and that people with low vitamin D levels are at higher risks of developing depression [10,11,12]. Moreover, other studies have also found that vitamin D supplements can reduce depressive symptoms in depressed patients [9,13]. Therefore, vitamin D is expected to be an effective means of depression treatment.

Vitamin D receptors are also widely present in sleep-related brain regions: 1alpha-hydroxylase, a regulatory enzyme of vitamin D, is also widely present in sleep-associated brain areas, including the hypothalamus [14]. The central circadian pacemaker is located in the hypothalamic suprachiasmatic nucleus (SCN) [15]. The SCN outputs signals that induce melatonin synthesis in the pineal gland under dim light. Vitamin D has been proposed to have an important role in regulating melatonin [16]. Melatonin is a hormone involved in regulating circadian rhythms and sleep, and vitamin D levels are also regulated by sunlight exposure. Since sunlight also affects circadian rhythms, this further suggests its relevance in the regulation of sleep [17]. Clinical studies also found that people who are deficient in vitamin D are more likely to develop sleep disturbances [18,19,20,21]. It is well known that insomnia is a core symptom of depression and also an independent risk factor for depression [22]. Studies have suggested that insomnia increases the risk of depression and anxiety, which, in turn, increases the risk of sleep disturbances [23,24]. So, it is very reasonable to assume that there is a close link between sleep disturbances and depression. In this study, we further investigated whether sleep disturbances affect the association between vitamin D and depressive symptoms. Vitamin D is obtained via sunlight, diet, and dietary supplements, and the compound leaves the body with no harmful effects if it is not excessively consumed. If the effects of vitamin D could be identified, then it would be possible to introduce more interventions earlier.

## 2. Materials and Methods

### 2.1. Patients

**Figure 1:**
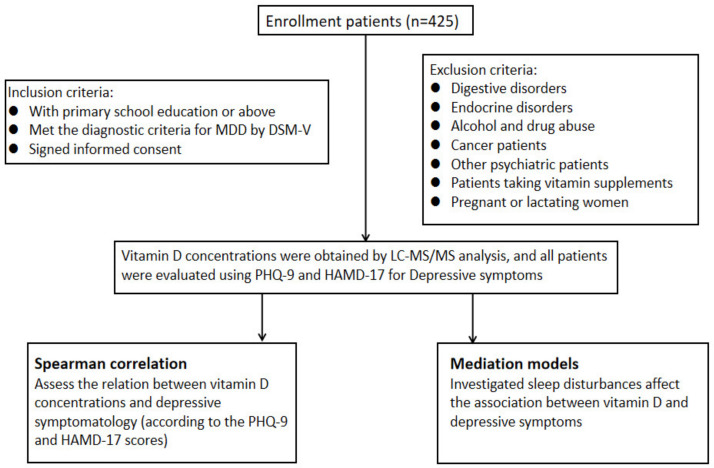
Flowchart representing the procedure of study.

A total of 425 patients with depression aged 18–55 years from Wuhan in Hubei Province were retrieved from the ESCID (Early Warning System and Comprehensive Intervention for Depression) database, which includes data from April 2019 to April 2021. The research was approved by the Ethics Committee of Renmin Hospital of Wuhan University (approval no.WDRY2020-K191). These patients were with primary school education or above. Depression was diagnosed by an experienced psychiatrist and met the diagnostic criteria for major depressive disorder (MDD) recommended by the fifth edition of the Diagnostic and Statistical Manual of Mental Disorders. Standardized interviews were performed to collect sociodemographic characteristics and medical histories. General physical examinations (such as measuring weight and height), scale assessments (Patient Health Questionnaire-9 (PHQ-9), the Hamilton Rating Scale for Depression (HAMD-17), and laboratory tests (e.g., blood sampling) were also performed. All participants signed informed consent. Exclusion criteria included the following: (1) digestive disorders; (2) endocrine disorders; (3) alcohol and drug abuse; (4) cancer patients; (5) other psychiatric patients; (6) patients taking vitamin supplements; (7) pregnant or lactating women (as shown in Figure 1).

### 2.2. Measures

The presence of current depressive symptoms was assessed using PHQ-9 and HAMD-17 (as shown in Table S1); PHQ-9 was assessed by the patients, while the HAMD-17 scale was assessed by two psychiatrists with extensive experience. PHQ-9 comprises 9 items, and the sum score ranges from 0 to 27, with higher total scores reflecting more depressive symptoms. We computed a modified PHQ-9 sum score by removing item 3 (i.e., sleep problems) from the original PHQ-9 total score. HAMD-17 comprises 17 items, and the sum score ranges from 0 to 53. We computed a modified HAMD-17 sum score by removing items 4–6 (i.e., sleep problems) from the original HAMD-17 total score. The scales were completed on the day of blood collection. A blood sample of 5 mL was collected using a blood collection vessel containing inert separation glue and coagulant on an empty stomach in the morning. It then stood for 30 min and was centrifuged at 2000× *g* for 15 min. The supernatant was taken and sent to the laboratory department of the hospital for liquid chromatography–tandem mass spectrometry (LC-MS/MS) analysis [25].

### 2.3. Statistical Analyses

Demographical characteristics and clinical variables were analyzed using descriptive statistics. Using the Shapiro–Wilks test on vitamin D concentrations and scores from assessment scales, we determined that there was a non-normal distribution. Spearman correlation coefficients were chosen to assess the relation between vitamin D concentrations and depressive symptomatology (according to the PHQ-9 and HAMD-17 scores) using R version 4.1.0 (R Project for Statistical Computing). The correlation calculation is conducted using the corr.test() function in the psych package, and significance levels were set at 0.05, 0.01, 0.001 (two-tailed).

The GLM Mediation Model in the Medmod module for data analysis in Jamovi 2.2.5 was used to analyze the mediation models for sleep disturbances; the method was based on the mediation method with 5000 bootstrap bias-corrected 95% confidence intervals, recommended by Hayes [26] as measures of indirect effects. The indirect effect can be considered statistically significant if the 95% confidence interval does not include zero [27]. Two mediation models were constructed in this study: in the first model, item 3 of PHQ-9 was selected as the mediator variable, vitamin D was the independent variable, and the total score of PHQ-9 after adjustment (PHQ-8 sum scores) was the dependent variable; in the second model, items 4, 5, and 6 of HAMD-17 were selected as the mediating variables, vitamin D as the independent variable, and the adjusted HAMD-17 total score (HAMD-14 sum scores) as the dependent variable. In the two models, three variables (gender, age, and BMI) were treated as covariates to control for the potential confounders. A *p* value < 0.05 was considered statistically significant.

## 3. Results

### 3.1. Participant Characteristics and Scores of Scales

The characteristics of the participants are shown in Table 1. The mean serum 25 (OH)D concentration in the sample (14.5 ng/mL; SD: 6.9 ng/mL) reflects vitamin D insufficiency [28]. As shown in Table 1, the study population (n = 425) included 105 males (24.7%) and 320 females (75.3%). Most of the patients were students (67.9%), had first-episode depression (61.9%), unmarried, divorced, or separated (67.5%), married or cohabiting (32.5%), and had an undergraduate education (82.6%). The Han people constituted most of the patients (92.5%); 16.5% were from rural areas, and 83.5% were from urban areas. Their mean (SD) age was 23 (5.3), and the mean (SD) BMI was 20.7 (3.0). The mean (SD) scores for P3, H4, H5, H6, PHQ-8, and HAMD-14 were 2.1 (0.9), 1.1 (0.8), 1.0 (0.7), 0.9 (0.8), 14.7 (4.9), and 16.8 (5.8), respectively.

### 3.2. The Correlation Test of Scale Scores with Vitamin D Concentration

**Figure 2:**
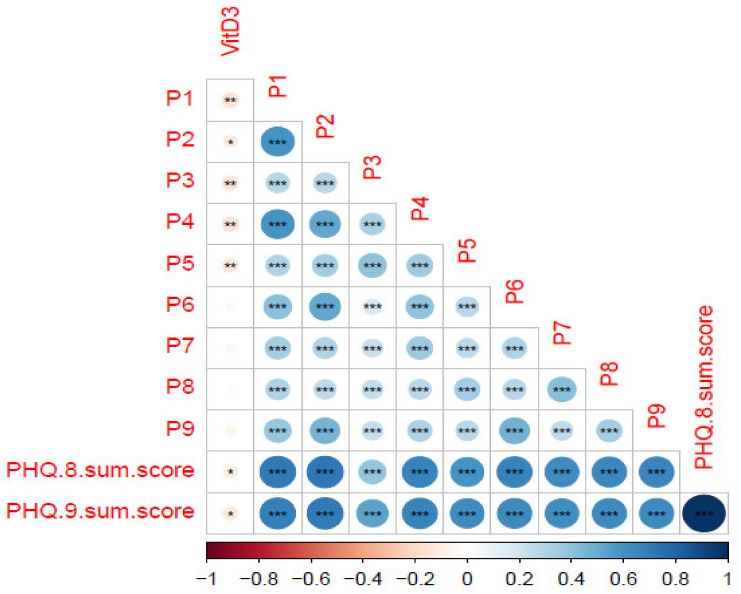
Summarizes the trilateral associations between PHQ-9 scores and vitamin D concentrations. PHQ-9, Patient Health Questionnaire-9. PHQ-8 sum score, without item 3 representing sleep problems. Use Corr. Test to calculate Spearman correlation, FDR correction, and Corrplot for visualization. The color represents the correlation, and the *p* value is significant on the annotation chart. *** = *p* < 0.001, ** = *p* < 0.01, * = *p* < 0.05.

**Figure 3:**
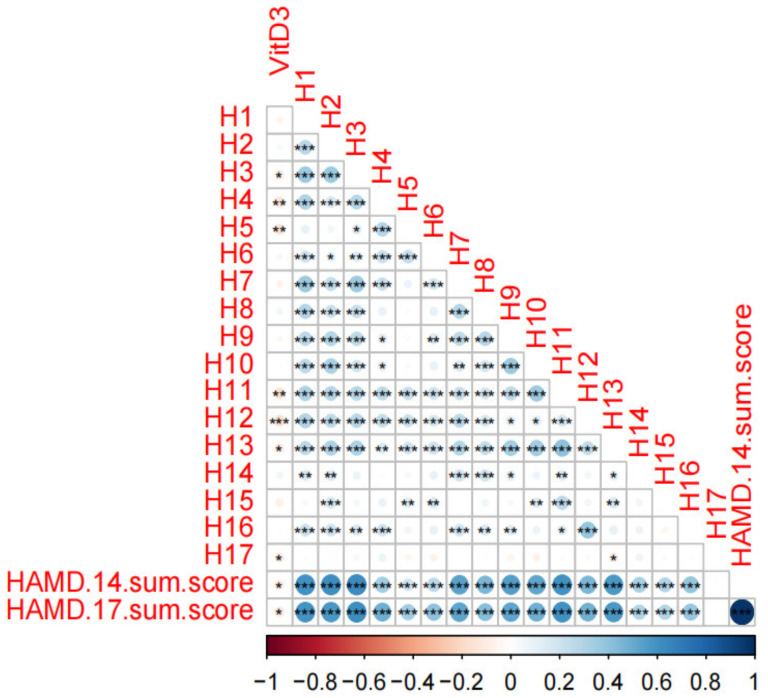
Summary of the trilateral associations between HAMD-17 scores and vitamin D concentrations. HAMD-17, Hamilton Rating Scale for Depression. HAMD-14 sum score, without items 4, 5, and 6 representing sleep problems. Use Corr. Test to calculate Spearman correlation, FDR correction, and Corrplot for visualization. The color represents the correlation, and the *p* value is significant on the annotation chart. *** = *p* < 0.001, ** = *p* < 0.01, * = *p* < 0.05.

As shown in Figure 2, in the correlation test of PHQ-9 items and sum scores with vitamin D concentration, vitamin D concentration was significantly correlated with P1, P2, P3, P4, P5, PHQ-8 sum score, and PHQ-9 sum score (*p* < 0.05). Vitamin D was significantly associated with multiple depressive symptoms of PHQ-9, including sleep disturbance-related item P3 (trouble falling or staying asleep or sleeping too much). As shown in Figure 3, vitamin D concentration was significantly correlated with H3, H4, H5, H11, H12, H13, H17, HAMD-14 sum score, and HAMD-17 sum score (*p* < 0.05) in the correlation test between each item and sum score of HAMD-17 with vitamin D concentration. Vitamin D was also significantly associated with depressive symptoms of HAMD-17, including sleep disturbances-related items H4 (trouble falling or staying asleep or sleeping too much), H5 (insomnia: middle of the night), H6 (insomnia: early hours of the morning).

### 3.3. The Mediation Models for Sleep

In our mediation effects model, the total effect of vitamin D on depressive symptoms is “c”, and the effect of vitamin D on sleep disturbances is “a”. The influence of sleep disturbances on depressive symptoms is “b”, “ab” is the mediating effect on sleep disturbances, and “c′” is the direct effect of vitamin D on depressive symptoms. Their relationship is as follows:

c = c′ + ab

If c ≠ 0, a ≠ 0, b ≠ 0 are significant, this indicates that there is an indirect effect. Meanwhile, if c′ ≠ 0 is significant, there is a partial indirect effect. If c′ = 0, there is a full indirect effect, and the direct effect is zero.

**Figure 4:**
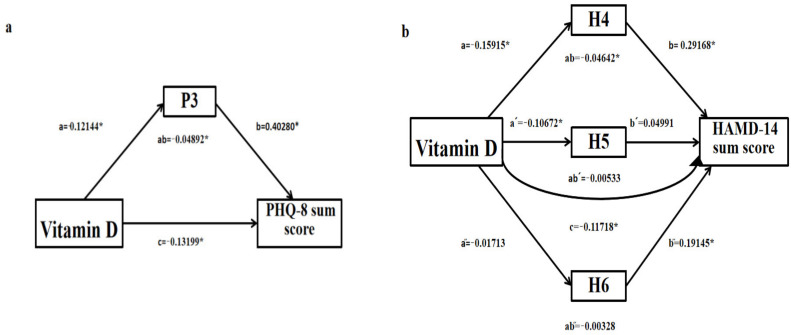
(**a**) Item 3 of PHQ-9 was selected as the mediator variable; (**b**) items 4, 5, and 6 of HAMD-17 were selected as the mediating variables). Mediation model showing both the total effect of vitamin D concentrations on depressive symptoms (path coefficient c) and the indirect effect of vitamin D concentrations on depressive symptoms mediated via sleep disturbances (as measured using modified PHQ-8 sum score and HAMD-14 sum score) (path coefficient ab) in the study sample. The figure depicts the unstandardized path coefficients (a, b, c, and ab). PHQ-8 sum score, without item 3 representing sleep disturbances. HAMD-14 sum score, without items 4, 5, and 6 representing sleep disturbances. * *p* < 0.05.

As shown in Figure 4a, in the first model with item 3 of PHQ-9 (used as the mediator variable), the effect of vitamin D concentrations on item 3 in this model (a = −0.12144; *p* = 0.022) and the effect of item 3 on PHQ-8 sum scores were significant (b = 0.40280; *p* < 0.001). The direct effect of vitamin D concentrations on PHQ-8 sum scores (c′ = −0.08308; *p* = 0.085, 95% CI [−0.12748, 0.00673]) was not significant. In addition, the indirect effect of item 3 in this model (ab = −0.04892; *p* = 0.031) and the total effect of vitamin D concentrations on PHQ-8 sum scores were significant (c = −0.13199; *p* = 0.010) (Table S2 for details). As shown in Figure 4b, in the second model with items 4, 5, and 6 of HAMD-17 (as the mediator variable), the indirect effects of item 5 (ab′ = −0.00533; *p* = 0.401, 95% CI [−0.01656, 0.00454]) and item 6 (ab″ = −0.00328; *p* = 0.735, 95% CI [−0.01909, 0.01336]) were not significant; only item 4 had a significant mediating effect (ab = −0.04642; *p* = 0.007). Also, the effect of vitamin D concentrations on item 4 in this model (a = −0.15915; *p* = 0.002) and the effect of item 4 on HAMD-14 sum scores were significant (b = 0.29168; *p* < 0.001). The direct effect of vitamin D concentrations on HAMD-14 sum scores (c′ = −0.06430; *p* = 0.197, 95% CI [−0.13592, 0.02804]) was not significant. The total effect of vitamin D concentrations on HAMD-14 sum scores was significant (c = −0.11718; *p* = 0.022) (Table S3 for details).

## 4. Discussion

The main objective of this study was to examine the trilateral association between serum vitamin D concentration, depressive symptoms, and sleep disturbances in patients with depression in China and whether vitamin D affects depressive symptoms through sleep disturbances. As a neurosteroid hormone that can cross the blood–brain barrier, vitamin D acts by binding to the VDR and its activating enzyme, 1ahydroxylase, which are broadly present in neuronal and glial cells of the human brain, suggesting that vitamin D has an important role in regulating cognitive, emotional, and sleep processes [14]. Our study confirms that vitamin D was significantly associated with multiple depressive symptoms, and sleep disturbances have a small significant effect on vitamin D and depressive symptoms, with difficulty falling asleep, in particular, acting as a significant mediator of vitamin D’s effect on depressive symptoms. These findings could explain the increase in sleep disturbances and depressive symptoms noted in patients with vitamin D deficiency and may suggest that vitamin D status is one of the modulators of the circadian rhythm.

Although the exact physiological mechanism between vitamin D, sleep disturbances, and depression is not fully understood, there are some possible hypotheses. First and most significant is that vitamin D has an important role in regulating 5-HT and melatonin. Some researchers found two distinct vitamin D response elements (VDREs) present in the regulatory regions of both tryptophan hydroxylase 2 (TPH2) and tryptophan hydroxylase 1 (TPH1), responsible for the conversion of tryptophan into serotonin [29]. The hormones act differently, with TPH2 being transcriptionally activated in the brain and TPH1 repressed in tissues outside the blood–brain barrier [30]. An adequate level of vitamin D in the brain increases the expression of TPH2, which is necessary for producing adequate serotonin, and, in turn, positively affects mood, cognition, and behavior. In peripheral tissues outside the blood–brain barrier, serum 25(OH)D binding to vitamin D response elements (VDREs) on TPH genes can inhibit the expression of TPH1 so that less serotonin is produced [30]. Melatonin regulation is a potentially important factor in the effect of vitamin D in inhibiting peripheral 5-HT production. The pineal gland, a periventricular tissue located outside the blood–brain barrier, converts the 5-HT produced via the expression of TPH1 into melatonin to improve sleep at night [31]. Studies have shown that both 25(OH)D and the active form 1,25(OH)2D follow a 24 h pattern [29]; the levels are the highest at noon (more sunlight) and lowest in the evening (less sunlight). During the day, when vitamin D levels are high, TPH2 expression increases, allowing the brain to produce enough serotonin to improve mood and cognition; at night, when serum vitamin D decreases, the expression of TPH1 increases in the pineal gland and promotes the production of 5-HT, which is then converted into melatonin to improve sleep. Good sleep is also conducive to mood and cognition during waking hours. This suggests the importance of taking vitamin D supplements at a reasonable time and explains why some studies of vitamin D supplementation have shown no benefit for depressive symptoms [32,33].

Secondly, the effects of vitamin D on sleep and mood may also be related to inflammatory immunity. As an immunomodulatory molecule, vitamin D is involved in the downregulation of inflammatory markers (such as tumor necrosis factor α (TNF-α), etc.). In the absence of vitamin D, these inflammatory markers are elevated, which can negatively affect sleep and mood [34]. Thirdly, vitamin D is also potentially linked to the gut microbiome [35]. Studies have found that vitamin D may restore intestinal barrier damage through its immunomodulatory activity [36], thereby promoting the growth of intestinal microbial balance [37]. Changes in the gut microbiota may influence the development of neuropsychiatric disorders, including mood disorders [38]. In addition, this relationship may also be influenced by other factors, such as obesity [39] and light [40,41]. Low vitamin D levels in obese patients may be due to insufficient vitamin D intake, increased fat or muscle mass, or genotypic variations in vitamin D metabolizing enzymes [42,43]. Both obesity and vitamin D deficiency contribute to chronic low-grade inflammation, which is thought to contribute to the development of depression and sleep disturbances [44]. In fact, the possibility of reverse causality should also be noted. People with depression and obesity may suffer from vitamin D deficiency due to image problems, social withdrawal, anhedonia, pathological fatigue, and prolonged avoidance of outdoor activities and sun exposure [45]. In addition, low-quality diets and metabolic disorders associated with obesity and depression can increase the risk of vitamin D deficiency [46]. Vitamin D synthesis depends on light duration. Light also affects circadian rhythms via SCN, which affects mood and sleep. Given the direct effects of light on mood and sleep, it has been well used in winter and other depression and circadian sleep disturbances [47].

This study has a few limitations. First, this is a cross-sectional study and cannot account for causal effects. Second, we did not include normal controls, and we need to add longitudinal studies with normal controls in future studies to clarify the relationship between vitamin D, sleep disturbances, and depressive symptoms. Third, there were some missing data, such as dietary intake, medication status, and sunlight exposure. How to ensure the quantification and consistency of these data is a problem we are considering, and we will try our best to improve it in subsequent research. The fourth limitation is that the relationship between vitamin D, sleep disturbances, and depressive symptoms could be reciprocal, where sleep disturbances and depressive symptoms may lead to vitamin D deficiency by affecting the duration of light exposure, vitamin D metabolism, or even vitamin D uptake. Another major limitation is that there is a lack of specific sleep scales to assess sleep disturbances, and sleep quality was only based on subjective assessment. Although a previous study has used similar methods to assess sleep disturbances [48], it has been insufficient. So, specific sleep scales are needed, such as the Pittsburgh Sleep Quality Index (PSQI) or the Insomnia Severity Index (ISI), and a methodology that may need validation using more objective means, such as actigraphy or polysomnography. The use of objective measurement tools in future studies can be analyzed and compared with the data of specific sleep scales. Finally, part of the time period when we recruited patients coincided with the COVID-19 pandemic, and we cannot fail to note the impact of the pandemic. Therefore, follow-up studies need to use the data before and after the pandemic for comparison: more comprehensive large-scale studies are needed to better elucidate the role of sleep disturbances in vitamin D and depressive symptoms.

Sleep disturbances impact a person’s quality of life. However, despite their frequency and importance, such conditions often go unnoticed since all patients do not clearly manifest fully expressed symptoms. Therefore, sleep disturbances in these patients should be considered by healthcare providers as one of the challenging problems, and early detection and intervention to improve sleep should be necessary. If we could prevent or improve sleep disturbances through diet or simply increase sun exposure, that could be a very socially beneficial thing and one that most people could more easily accept.

## 5. Conclusions

Our study found that sleep disturbances (especially difficulty falling asleep) are mediators between vitamin D and depressive symptoms. Vitamin D may ameliorate depressive symptoms by regulating sleep via regulating 5-HT and melatonin, but the specific mechanism is not clear. Previous studies have shown that the photoperiod and levels of melatonin and vitamin D play an important role in regulating the sleep rhythm of humans, suggesting the need for vitamin D supplementation at a reasonable time is very important. Our study reveals a preliminary relationship, but due to the limitations of observational studies, this conclusion cannot be taken as a final decision. But it can prompt us to conduct more in-depth studies.

## Figures and Tables

**Table 1:** Socio-demographic characteristics of participants (n = 425).

Variables	Frequency/Mean	Percentage (%)/SD
**Gender**		
Male	105	24.7
Female	320	75.3
**Relationship status**		
Single ^a^	287	67.5
Has a partner ^b^	138	32.5
**Occupation**		
Student	287	67.5
Unemployed	38	8.9
Employed	100	23.5
**Education level**		
High school or less	15	3.5
Undergraduate	351	82.6
Postgraduate or higher	59	13.9
**First episode**		
Yes	263	61.9
No	162	38.1
**Race**		
Ethnic Han	393	92.5
Minority	32	7.5
**Habitation**		
Urban	355	83.5
Rural	70	16.5
**Age**	23	5.3
**BMI**	20.7	3.0
**Vitamin D concentration (ng/mL)**	14.5	6.9
**P3** score	2.1	0.9
**H4** score	1.1	0.8
**H5** score	1.0	0.7
**H6** score	0.9	0.8
**PHQ-8** sum score	14.7	4.9
**HAMD-14** sum score	16.8	5.8
**PHQ-9** sum score	16.9	5.4
**HAMD-17** sum score	19.7	6.6

^a^ Included unmarried, divorced, or separated participants. ^b^ Included married or cohabiting participants. BMI, Body Mass Index; PHQ-9, Patient Health Questionnaire-9; HAMD-17, Hamilton Rating Scale for Depression; P3, item 3 of PHQ-9 (trouble falling or staying asleep, or sleeping too much); H4, item 4 of HAMD-17 (trouble falling or staying asleep, or sleeping too much); H5, item 5 of HAMD-17 (insomnia: middle of the night); H6, item 6 of HAMD-17 (insomnia: early hours of the morning); PHQ-8, PHQ-9 without P3; HAMD-14, HAMD-17 without H4, H5, and H6.

## Data Availability

Data are available upon request from the authors.

## Supplementary Materials

The following supporting information can be downloaded at: https://www.mdpi.com/article/10.3390/brainsci13111501/s1, Table S1: PHQ-9 and HAMD-17 items; Table S2: Path Plot of PHQ-9; Table S3: Path Plot of HAMD-17.
