# Supplementary material for: The Mediation Role of Sleep Disturbances between Vitamin D and Depressive Symptoms: A Cross-Sectional Study
Source: Brain Sci. 2023 Oct 24;13(11):1501. doi: 10.3390/brainsci13111501 (PMC10669134; doi:10.3390/brainsci13111501)
Supplement: Supplementary file 1 [file brainsci-13-01501-s001.zip › Table S1. PHQ-9 and HAMD-17 items.pdf]

PHQ-9

P1: Little interest or pleasure in doing things

P2: Feeling down, depressed, or hopeless

P3: Trouble falling or staying asleep, or sleeping too much

P4: Feeling tired or having little energy

P5: Poor appetite or overeating

P6: Feeling bad about yourself

P7: Trouble concentrating on things

P8: Moving or speaking so slowly that other people could have noticed– Or so fidgety or restless that you have been moving a lot more than usual

P9: Thoughts that you would be better off dead, or thoughts of hurting yourself in some way

HAMD-17

H1: Depressed mood (sadness, hopeless, helpless, worthless)

H2: Feelings of guilt

H3: Suicide

H4: Insomnia: early in the night

H5: Insomnia: middle of the night

H6: Insomnia: early hours of the morning

H7: Work and activities

H8: Retardation

H9: Agitation

H10: Anxiety psychic

H11: Anxiety somatic

H12: Somatic symptoms gastrointestinal

H13: General somatic symptoms

H14: Genital symptoms

H15: Hypochondriasis

H16: Loss of weight

H17: Insight
