# Supplementary material for: The Mediation Role of Sleep Disturbances between Vitamin D and Depressive Symptoms: A Cross-Sectional Study
Source: Brain Sci. 2023 Oct 24;13(11):1501. doi: 10.3390/brainsci13111501 (PMC10669134; doi:10.3390/brainsci13111501)
Supplement: Supplementary file 1 [file brainsci-13-01501-s001.zip › Table S2. Path Plot of PHQ-9.pdf]

Mediation

Indirect and Total Effects

Type	Effect	Estimate	SE	95% C.I. (a) Lower	Upper	β	z	p
Indirect	VitD3 ⇒ P3 ⇒ PHQ-8 sum score	-0.03495	0.01618	-0.06862	-0.00478	-0.04892	-2.161	0.031
	sex1 ⇒ P3 ⇒ PHQ-8 sum score	0.30226	0.24262	-0.17122	0.79591	0.02657	1.246	0.213
	age ⇒ P3 ⇒ PHQ-8 sum score	0.00909	0.01685	-0.02445	0.04280	0.00974	0.540	0.589
	BMI ⇒ P3 ⇒ PHQ-8 sum score	0.03168	0.03573	-0.04042	0.09997	0.01952	0.887	0.375
Component	VitD3 ⇒ P3	-0.01613	0.00706	-0.02993	-0.00212	-0.12144	-2.285	0.022
	P3 ⇒ PHQ-8 sum score	2.16708	0.25901	1.69071	2.69402	0.40280	8.367	< .001
	sex1 ⇒ P3	0.13948	0.10986	-0.07599	0.35231	0.06597	1.270	0.204
	age ⇒ P3	0.00420	0.00764	-0.01090	0.01888	0.02417	0.549	0.583
	BMI ⇒ P3	0.01462	0.01629	-0.01822	0.04554	0.04845	0.898	0.369
Direct	VitD3 ⇒ PHQ-8 sum score	-0.05936	0.03442	-0.12748	0.00673	-0.08308	-1.725	0.085
	sex1 ⇒ PHQ-8 sum score	-0.73800	0.51373	-1.75458	0.29034	-0.06488	-1.437	0.151
	age ⇒ PHQ-8 sum score	0.08178	0.03964	0.00267	0.15597	0.08756	2.063	0.039
	BMI ⇒ PHQ-8 sum score	-0.05247	0.07037	-0.19656	0.08352	-0.03232	-0.746	0.456
Total	VitD3 ⇒ PHQ-8 sum score	-0.09431	0.03650	-0.16585	-0.02277	-0.13199	-2.584	0.010
	sex1 ⇒ PHQ-8 sum score	-0.43575	0.58623	-1.58474	0.71325	-0.03831	-0.743	0.457
	age ⇒ PHQ-8 sum score	0.09087	0.04495	0.00277	0.17898	0.09729	2.022	0.043
	BMI ⇒ PHQ-8 sum score	-0.02079	0.07949	-0.17659	0.13502	-0.01280	-0.261	0.794

Note. Confidence intervals computed with method: Bootstrap percentiles

Note. Betas are completely standardized effect sizes
