# Supplementary material for: The Mediation Role of Sleep Disturbances between Vitamin D and Depressive Symptoms: A Cross-Sectional Study
Source: Brain Sci. 2023 Oct 24;13(11):1501. doi: 10.3390/brainsci13111501 (PMC10669134; doi:10.3390/brainsci13111501)
Supplement: Supplementary file 1 [file brainsci-13-01501-s001.zip › Table S3. Path Plot of HAMD-17.pdf]

Indirect and Total Effects

Type	Effect	Estimate	SE	95% C.I. (a)		β	z	p
				Lower	Upper			
Indirect	VitD3 ⇒ H4 ⇒ HAMD-14 sum score	-0.03848	0.01437	-0.06880	-0.01278	-0.04642	-2.6777	0.007
	VitD3 ⇒ H5 ⇒ HAMD-14 sum score	-0.00442	0.00526	-0.01656	0.00454	-0.00533	-0.8401	0.401
	VitD3 ⇒ H6 ⇒ HAMD-14 sum score	-0.00272	0.00804	-0.01909	0.01336	-0.00328	-0.3380	0.735
	age ⇒ H4 ⇒ HAMD-14 sum score	-5.53e−4	0.01495	-0.02898	0.03033	-5.10e−4	-0.0370	0.971
	age ⇒ H5 ⇒ HAMD-14 sum score	0.00344	0.00480	-0.00406	0.01496	0.00318	0.7168	0.473
	age ⇒ H6 ⇒ HAMD-14 sum score	0.03191	0.01364	0.00954	0.06219	0.02944	2.3385	0.019
	BMI ⇒ H4 ⇒ HAMD-14 sum score	0.05456	0.02971	4.23e-6	0.11838	0.02897	1.8363	0.066
	BMI ⇒ H5 ⇒ HAMD-14 sum score	-0.00477	0.00796	-0.02374	0.00936	-0.00253	-0.5992	0.549
	BMI ⇒ H6 ⇒ HAMD-14 sum score	-0.00890	0.01718	-0.04291	0.02650	-0.00472	-0.5181	0.604
	sex1 ⇒ H4 ⇒ HAMD-14 sum score	-0.09903	0.21682	-0.54931	0.32350	-0.00750	-0.4567	0.648
	sex1 ⇒ H5 ⇒ HAMD-14 sum score	0.03850	0.06023	-0.05591	0.18765	0.00292	0.6391	0.523
	sex1 ⇒ H6 ⇒ HAMD-14 sum score	-0.21588	0.14161	-0.52493	0.03635	-0.01636	-1.5245	0.127
Component	VitD3 ⇒ H4	-0.01952	0.00642	-0.03180	-0.00695	-0.15915	-3.0405	0.002
	H4 ⇒ HAMD-14 sum score	1.97179	0.34029	1.32164	2.64320	0.29168	5.7944	< .001
	VitD3 ⇒ H5	-0.01147	0.00555	-0.02288	-7.47e−4	-0.10672	-2.0672	0.039
	H5 ⇒ HAMD-14 sum score	0.38487	0.37472	-0.35320	1.10678	0.04991	1.0271	0.304
	VitD3 ⇒ H6	-0.00197	0.00571	-0.01296	0.00956	-0.01713	-0.3446	0.730
	H6 ⇒ HAMD-14 sum score	1.38196	0.34182	0.71225	2.04201	0.19145	4.0430	< .001
	age ⇒ H4	-2.80e−4	0.00747	-0.01427	0.01503	-0.00175	-0.0375	0.970
	age ⇒ H5	0.00895	0.00628	-0.00255	0.02207	0.06368	1.4245	0.154
	age ⇒ H6	0.02309	0.00775	0.00847	0.03871	0.15378	2.9799	0.003
	BMI ⇒ H4	0.02767	0.01394	2.27e-6	0.05489	0.09931	1.9845	0.047
	BMI ⇒ H5	-0.01239	0.01262	-0.03617	0.01321	-0.05074	-0.9821	0.326
	BMI ⇒ H6	-0.00644	0.01214	-0.02959	0.01839	-0.02468	-0.5306	0.596
	sex1 ⇒ H4	-0.05022	0.10727	-0.26330	0.16081	-0.02573	-0.4682	0.640
	sex1 ⇒ H5	0.10003	0.08869	-0.07327	0.28081	0.05845	1.1278	0.259
	sex1 ⇒ H6	-0.15621	0.09284	-0.33719	0.02789	-0.08545	-1.6825	0.092
Direct	VitD3 ⇒ HAMD-14 sum score	-0.05330	0.04133	-0.13592	0.02804	-0.06430	-1.2897	0.197
	age ⇒ HAMD-14 sum score	0.00380	0.05033	-0.10176	0.09774	0.00351	0.0755	0.940
	BMI ⇒ HAMD-14 sum score	-0.09375	0.09229	-0.27642	0.08956	-0.04978	-1.0159	0.310

Note. Confidence intervals computed with method: Bootstrap percentiles

Note. Betas are completely standardized effect sizes

Indirect and Total Effects

Type	Effect	Estimate	SE	95% C.I. (a)		β	z	p
				Lower	Upper			
	sex1 ⇒ HAMD-14 sum score	-0.01754	0.62904	-1.24508	1.20693	-0.00133	-0.0279	0.978
Total	VitD3 ⇒ HAMD-14 sum score	-0.09892	0.04334	-0.18386	-0.01398	-0.11718	-2.2827	0.022
	age ⇒ HAMD-14 sum score	0.03860	0.05337	-0.06600	0.14320	0.03498	0.7232	0.470
	BMI ⇒ HAMD-14 sum score	-0.05287	0.09438	-0.23784	0.13211	-0.02756	-0.5602	0.575
	sex1 ⇒ HAMD-14 sum score	-0.29395	0.69598	-1.65805	1.07015	-0.02187	-0.4224	0.673

Note. Confidence intervals computed with method: Bootstrap percentiles

Note. Betas are completely standardized effect sizes
